# Injuries and Diseases of the Genital and Urinary Organs

**Published:** 1896-06

**Authors:** 


**Injuries and Diseases of the Genital and Urinary Organs.** By Henry Morris, M.B. Pp. xvi., 478. London: Cassell and Company, Limited. 1895.

Mr. Henry Morris's previous work on surgical diseases of the kidneys was so good that we opened the present book with considerable expectations, which have not been altogether fulfilled by a careful perusal of it. A great many subjects are touched upon, but none of them seem to be dealt with in a thorough manner. The style of the book is dull, and partakes too much of the nature of a dictionary, while at the same time there is a seeming want of completion in many of the subjects treated. This is no doubt owing largely to the fact that no details are given of any operation recommended in the book, the reason given in the preface being the necessity of keeping the descriptions as brief as possible; but we think that by this omission the author has destroyed much of the usefulness of the work. Thus, in a work on diseases of the genital and urinary organs, surely we should look for some account of lithotomy and lithotrity, and for some description of the best method of removing an enlarged prostate gland; yet the work before us presents us with a mere outline of these operations, giving hardly any details, and simply signifying the particular operation most favoured by the author. These are serious omissions, and will render the book much less valuable as a work of reference than it otherwise would have been.

The author has taken care to bring the book up to date, and it presents us with an outline of the present knowledge of the diseases of the urinary organs in a condensed and handy form. It is evidently the outcome of a considerable personal experience, and bears evidence on every page of careful forethought and sound good sense. It is well illustrated with ninety-seven illustrations, many of which are entirely new and almost all of which are good.

